# The Effect of Plant-Derived Low-Ratio Linoleic Acid/α-Linolenic Acid on Markers of Glucose Controls: A Systematic Review and Meta-Analysis

**DOI:** 10.3390/ijms241814383

**Published:** 2023-09-21

**Authors:** Qiong Wang, Xingguo Wang

**Affiliations:** State Key Laboratory of Food Science and Technology, Collaborative Innovation Center of Food Safety and Quality Control in Jiangsu Province, School of Food Science and Technology, Jiangnan University, Wuxi 214122, China; wangqiong9595@163.com

**Keywords:** linoleic acid/α-linolenic acid, glucose markers, insulin, meta-analysis, randomized controlled trials

## Abstract

The objective of this meta-analysis was to examine the impact of a low-ratio linoleic acid/α-linolenic acid (LA/ALA) diet on the glycemic profile of adults. A comprehensive search was performed across four databases (Web of Science, Scopus, Embase, and PubMed) to evaluate the influence of the low-ratio LA/ALA. Relevant references were screened up until February 2023. Intervention effects were analyzed by calculating change values as weighted mean differences (WMD) and 95% confidence intervals (CI) using fixed-effects models. Additionally, subgroup analysis and meta-regression were employed to investigate potential sources of heterogeneity. Twenty-one randomized controlled trials (RCTs) were included, and the low-ratio LA/ALA diet had no significant effect on fasting blood sugar (FBS, WMD: 0.00 mmol/L, 95% CI: −0.06, 0.06, *p* = 0.989, I^2^ = 0.0%), insulin levels (WMD: 0.20 μIU/mL, 95% CI: −0.23, 0.63, *p* = 0.360, I^2^ = 3.2%), homeostatic model assessment insulin resistance (HOMA-IR, WMD: 0.09, 95% CI: −0.06, 0.23, *p* = 0.243, I^2^ = 0.0%), and hemoglobin A1c (HbA1c, WMD: −0.01%, 95% CI: −0.07, 0.06, *p* = 0.836, I^2^ = 0.0%). Based on subgroup analyses, it was observed that the impact of a low-ratio LA/ALA diet on elevated plasma insulin (WMD: 1.31 μIU/mL, 95% CI: 0.08, 2.54, *p* = 0.037, I^2^ = 32.0%) and HOMA-IR (WMD: 0.47, 95% CI: 0.10, 0.84, *p* = 0.012, I^2^ = 0.0%) levels exhibited greater prominence in North America compared to Asian and European countries. Publication bias was not detected for FBS, insulin, HOMA-IR, and HbA1c levels according to the Begg and Egger tests. Furthermore, the conducted sensitivity analyses indicated stability, as the effects of the low-ratio LA/ALA diet on various glycemic and related metrics remained unchanged even after removing individual studies. Overall, based on the available studies, it can be concluded that the low-ratio LA/ALA diet has limited impact on blood glucose-related biomarker levels.

## 1. Introduction

Diabetes is a prevalent chronic disease characterized by abnormalities in glucose metabolism, and its global incidence is on the rise. According to the International Diabetes Federation (IDF), the global prevalence of diabetes was estimated to be 10.5% (537 million individuals) in 2021, with projections indicating a rise to 12.2% (783 million individuals) by 2045 [1]. This alarming trend is not only evident in high-income countries, but the largest increases are observed in middle-income countries [2]. As a significant non-communicable disease, diabetes poses a substantial burden on public health [3]. Diabetes, associated with elevated fasting blood sugar (FBS) and insulin levels, disrupts the normal biological functioning, and significantly increases the risk of conditions such as retinopathy, coronary heart disease, renal failure, neuropathy, and various types of cancer [4,5,6,7].

Strategies such as dietary patterns, individual nutrients and lifestyle are effective in the prevention and management of diabetes [8]. The quality of fats and carbohydrates in the diet is more important than the quantity of these macronutrients. Polyunsaturated fatty acids (PUFAs), specifically n-3 PUFAs, have a significant impact on alleviating hyperglycemia and its associated complications [9,10,11]. These N-3 PUFAs comprise eicosapentaenoic acid (EPA), docosapentaenoic acid (DPA), and docosahexaenoic acid (DHA) derived from animal sources, as well as α-linolenic acid (ALA) sourced from plants. N-6 PUFAs, such as linoleic acid (LA), are derived from plants. Both ALA and LA are essential fatty acids (EFAs) that the human body needs, with the differentiation being based on the position of the initial double bond counted starting from the methyl end of the fatty acid (FA) molecule. LA and ALA share the same desaturase enzyme and have a competitive inhibitory relationship [12]. On the one hand, arachidonic acid (AA), a downstream product of LA, increases the biosynthesis of pro-inflammatory eicosanoids [13]. On the other hand, LA competes with ALA, inhibiting the conversion of ALA to n-3 long-chain PUFA to exert its biological activity [14]. Thus, the accomplishment of a balanced ratio of LA/ALA can help restoring the physiological equilibrium influenced by both genetic and environmental factors. Over the past few years, animal and cell culture studies have demonstrated that increasing ALA has beneficial effects on the prevention of type 2 diabetes mellitus (T2DM) through a variety of mechanisms, including alteration of cell membrane function, anti-inflammatory and antioxidant effects, insulin signaling, and control of glucose metabolism gene expression [15,16,17,18,19]. Epidemiologic evidence suggests that increasing ALA or decreasing LA dietary intake has a controlling effect on glucose and insulin levels. Numerous human intervention studies have explored the impacts of diets with varying LA/ALA ratios on factors such as FBS and insulin levels. However, the findings from these studies have been inconsistent and inconclusive. Some conflicting research has indicated that a low ration of LA/ALA: (a) was significantly associated with decreased levels of FBS [20], insulin [21], and hemoglobin A1c (HbA1c) [22]; (b) was significantly correlated with increased levels of FBS [23] and insulin [24]; and (c) did not have any significant effect on the levels of FBS, insulin, homeostatic model assessment insulin resistance (HOMA-IR), and HbA1c [25,26]. These variations can be attributed to factors such as sample size, duration of the intervention, and the type of intervention employed, including aspects like total energy intake, dietary intake of LA and ALA.

Although LA and ALA are some of the most common forms of PUFA supplementation, the complete metadata on the relationship between LA/ALA ratio and glycemia are still lacking. Consequently, the objective of this study was to comprehensively assess and compare the impacts of the low-ratio LA/ALA diet on FBS, insulin, HbA1c, and HOMA-IR using randomized controlled trials (RCTs) as the basis.

## 2. Methods

### 2.1. Search Strategy

To ensure that the study was conducted and reported in a systematic manner, we adhered to the guidelines outlined in the Preferred Reporting Items for Systematic Reviews and Meta-Analyses (PRISMA) statement. A comprehensive search was performed on four electronic databases, namely Web of Science, Scopus, Embase, and PubMed, up until February 2023. The search strategy employed is elaborated upon in Supplementary Table S1.

### 2.2. Inclusion Criteria

In order to be included in this review, studies had to meet specific criteria. These criteria included (1) focusing on the impact of LA/ALA ratio (in the form of plant oil, fat, and nuts) on various biological markers related to blood glucose control, such as FBS, insulin, HOMA-IR, and HbA1c; (2) the study participants consisted of individuals aged 18 years or older; (3) the intervention duration lasted at least two weeks; (4) the primary outcomes of articles reported sufficient information on baseline and final study; (5) the LA/ALA ratio was explicitly reported in the article, or it could be obtained by proper calculation; (6) to isolate the specific impact of the LA/ALA ratio on glucose, the included studies differentiated it from the influence of other dietary sources or interventions, such as fish oil, and conjugated linoleic acid (CLA), and physical activity programs. This approach allowed for a more focused analysis of the independent effects of the LA/ALA ratio on blood glucose control, minimizing confounding factors that could potentially influence the results.

### 2.3. Data Extraction

Two researchers independently conducted the data extraction based on the predefined inclusion criteria. The Cochrane Risk of Bias tool was employed to assess the methodological quality of the included studies. In case of any disputes, the original literature was re-evaluated to ensure accuracy and consistency. If there were any disagreements or conflicts during the study selection, a third reviewer was consulted to establish a consensus and resolve any discrepancies. The following data were extracted from each eligible study: the first author’s name, publication year, country where the study was conducted, type and duration of the intervention, total number of participants, participants characteristics (including disease, age, BMI, smoking status, and gender), protein, carbohydrates, and fat (saturated and monounsaturated FA, PUFA, LA, ALA) as percentage of total energy, LA/ALA ratio, and the mean and standard deviation (SD) changes in blood-glucose-related biomarkers at pre-treatment and post-treatment. In cases where multiple articles reported the same outcomes, preference was given to the article with the largest number of participants and longest duration for inclusion in the review.

### 2.4. Statistical Methods

Endnote software (Endnote X9.1) was employed to process the article, eliminating any duplicate entries. Subsequently, the screening and full text review were conducted using Microsoft Excel software (Microsoft Excel 16.0). To conduct the primary analysis, sensitivity analyses, and assess publication bias, we utilized STATA software (version 14.0, Stata Corp., College Station, TX, USA). Mean (SD) were extracted to perform a combined effect size analysis. Conversion formulas were utilized to calculate SD in situations where they were not directly provided [27]. If the standard deviation (SD) of the change was not provided in the trials, it was calculated using the formula: SD _change_ = square root [(SD _pre-treatment_)^2^ + (SD _post-treatment_)^2^ − (2R × SD _pre-treatment_ × SD _post-treatment_)] (R = 0.5). Moreover, when data were solely presented graphically, the relevant information was digitally extracted and quantified using the GetData Graph Digitizer software (GetData 2.25). The statistical significance of net changes was assessed using the weighted mean difference (WMD) with a 95% confidence interval (CI). The level of heterogeneity was classified as low (I^2^ ≤ 50%) or high (50% < I^2^), accordingly. Sensitivity analysis was conducted to investigate potential sources of heterogeneity, where each study was individually excluded to assess the impact of any specific study on the overall validity of the effect size. We utilized Begg’s tests, Egger regression tests, and visual calculations using funnel plots to evaluate possible publication bias. Furthermore, predetermined subgroup analyses were conducted to examine association between different subgroups and glucose. A *p* value below 0.05 was used to assess the statistical significance.

## 3. Results and Discussion

### 3.1. Study Selection and Description

**Figure 1:**
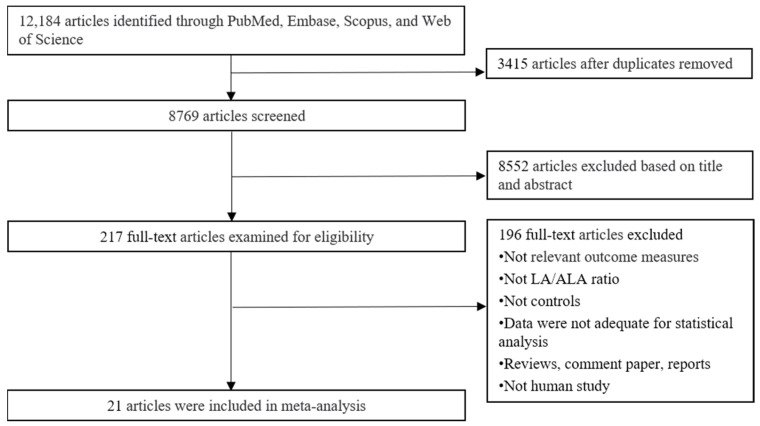
Screening flowchart of this study.

A total of 12,184 publications were initially searched from four databases, and 8769 articles were retained after eliminating duplicates. Subsequently, 217 articles were selected for full-text examination based on the titles and abstracts. Ultimately, 21 articles were included in this meta-analysis [22,23,24,28,29,30,31,32,33,34,35,36,37,38,39,40,41,42,43,44,45]. The screening process is depicted in Figure 1. Among the 21 articles that met the eligibility criteria, the effects of LA/ALA ratio on FBS were investigated in all 21 studies. Insulin was assessed in 17 studies, HOMA-IR in 10 studies, and HbA1c in 7 studies.

A detailed summary of the characteristics of the eligible trials is provided in Table 1, presenting the relevant information. The articles spanned from 1996 to 2020 in terms of publication dates. The study was conducted in several countries, including Iran, Netherlands, China, Denmark, UK, USA, Japan, Greece, Germany, Canada, India, Poland, Finland, Sweden. Among these, 6 studies were carried out in North America, 13 studies in Europe, 8 studies in Asia. A total of 17 articles used a parallel design and 4 articles used a crossover design. Sample sizes in the included studies ranged from 11 [38] to 243 [30]. The intervention duration ranged from 3 [23] to 48 [30] weeks. The eligible studies included participants of various ages, with an average age range within 22.8 [36] to 61.8 [38] years. The BMI of the participants ranged from 21.9 [35] to 39.6 [23]. The publications that met the eligibility criteria included participants of both genders, with two studies involving only women [22,44] and two only men [31,34]. Among the included trials, 11 studies specifically enrolled non-smoking subjects, whereas 7 included a combination of smoking and non-smoking subjects. Eligible study participants suffered from dyslipidemia [30,32,43,45], obesity [23,40], type 2 diabetes [24,37,38,42], metabolic syndrome [28,29], polycystic ovary syndrome [22,44], and non-alcoholic fatty liver [41], while healthy individuals were also included [31,33,34,35,36,39].

Supplementary Table S2 provides comprehensive details on dietary energy intake. The total energy intake, fat, protein, and carbohydrate supplementation (as a percentage of total energy) remained consistent. There was a significant difference in the total energy consumed by subjects in one study [32], a significant difference in the macronutrients consumed as a percentage of total energy by subjects in one study [42], and a significant difference in the fat consumed as a percentage of total energy by subjects in two studies [22,37]. Among the 21 studies analyzed, significant differences in PUFA were observed in 6 studies [28,31,33,37,40,42], MUFA and PUFA in 3 studies [35,36,39], and SFA, MUFA and PUFA in 2 studies [22,43]. Supplementation with flaxseed, canola oil, caper oil, hemp seed oil, and walnut can decrease the LA/ALA ratio. The low-ratio LA/ALA varied from 0.14 [28] to 9 [39], and the high-ratio LA/ALA varied from 4.3 [42] to 228.2 [41]. The range of dietary intake of LA ranged between 2.1% [28] and 18.1% [36] of total energy per day, while the ALA intake ranged from 0.21% [30] and 15.2% [28] of total energy per day.

The Cochrane Collaboration Risk of Bias Tool was utilized for the quality assessment of the included studies (Supplementary Table S3). Ten articles described randomized controlled methods. Ten articles had detailed methods describing allocation concealment. Most studies used single-blind or double-blind studies and concealed supplement allocation, except for 2 articles. Observational bias was not identified in most studies. Selective bias reporting was not described in most trails. There were no additional sources of bias identified across all studies.

### 3.2. Meta-Analysis Results

**Figure 2:**
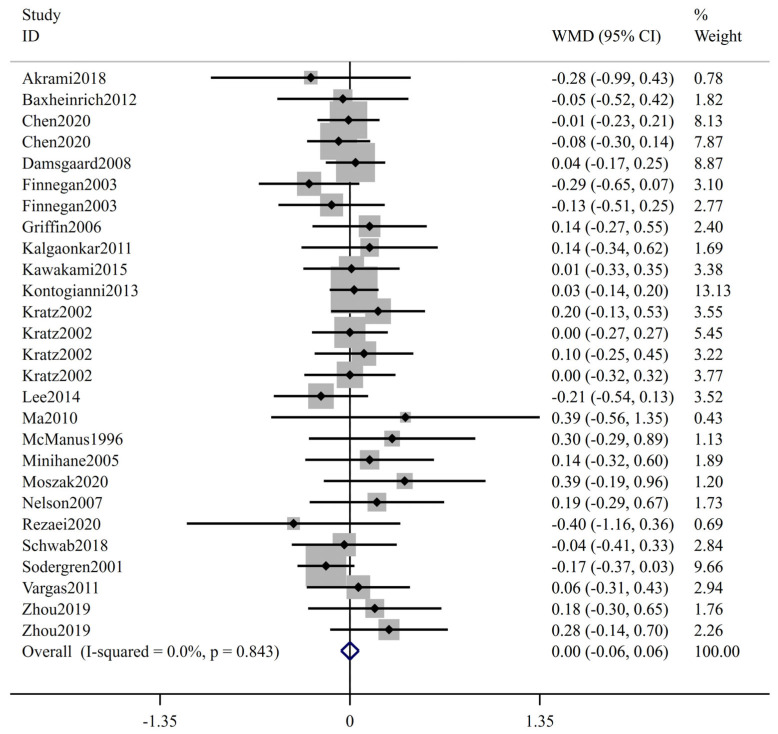
The effect of low-ratio LA/ALA on FBS. Refs. [22,23,24,28,29,30,31,32,33,34,35,36,37,38,39,40,41,42,43,44,45].

**Figure 3:**
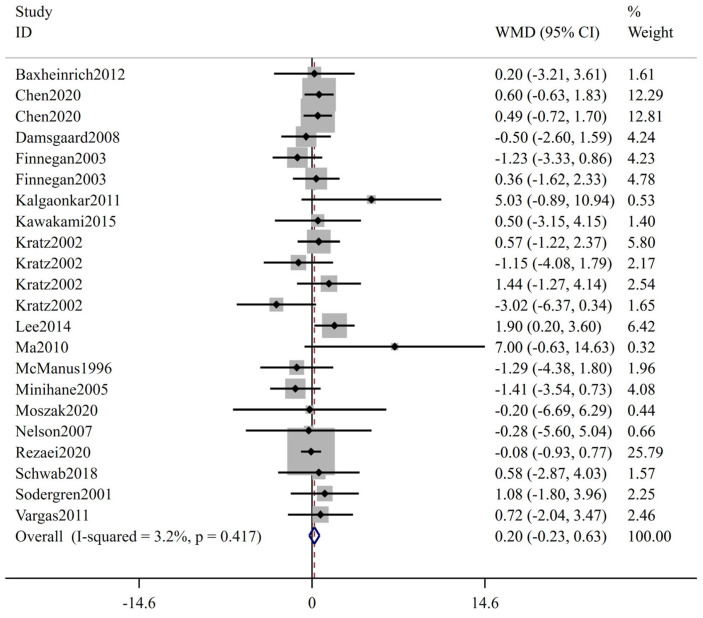
The effect of low-ratio LA/ALA on insulin. Refs. [22,23,24,29,30,31,32,34,36,37,38,39,40,41,42,43,44].

**Figure 4:**
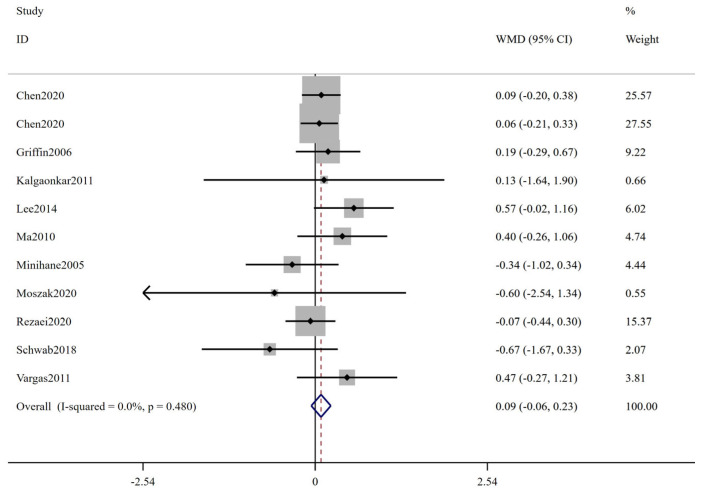
The effect of low-ratio LA/ALA on HOMA-IR. Refs. [22,23,24,30,33,37,39,41,42,44].

**Figure 5:**
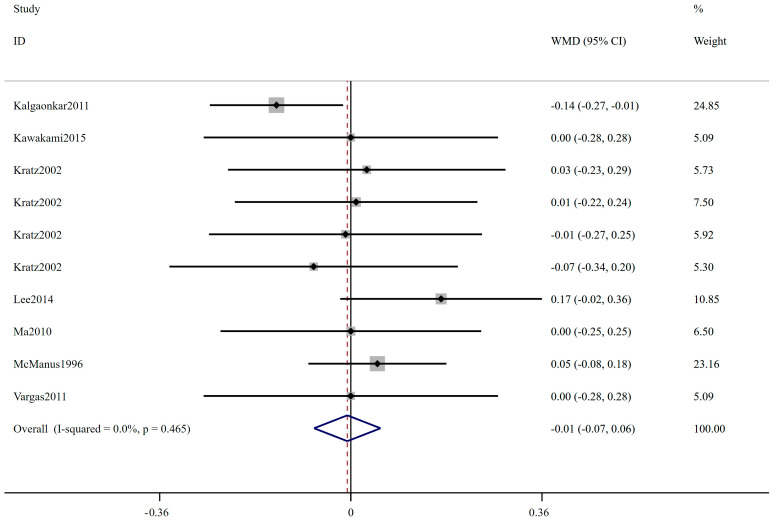
The effect of low-ratio LA/ALA on HbA1c. Refs. [22,24,34,36,37,38,44].

The forest plot of FBS is shown in Figure 2; 27 trials including 1415 participants (cases = 714, controls = 701) reported FBS as an outcome measure. According to the overall analysis from the fixed-effects models, the low-ratio LA/ALA diet did not lead to significant change on FBS (WMD: 0.00 mmol/L, 95% CI: −0.06, 0.06, *p* = 0.989). The I^2^ test indicated no statistically significant heterogeneity among the included studies (I^2^ = 0.0%, *p* = 0.843). Figure 3 displays the results for insulin, involving 22 trials with a total of 1096 participants (549 cases and 547 controls). Based on the pooled results from fixed-effects models, there was no significant change in insulin level following interventions with low-ratio LA/ALA diet (WMD: 0.20 μIU/mL, 95% CI: −0.23, 0.63, *p* = 0.360). The included trials exhibited non-significant heterogeneity (I^2^ = 3.2%, *p* = 0.417). Figure 4 illustrates the effect of low-ratio LA/ALA diet on HOMA-IR, involving eleven trials with a total of 736 participants (373 cases and 363 controls). The pooled findings indicated no significant decrease in HOMA-IR after consuming low-ratio LA/ALA diet (WMD: 0.09, 95% CI: −0.06, 0.23, *p* = 0.243). Heterogeneity was not significant, as indicated by the I^2^ value (I^2^ = 0.0%, *p* = 0.480). The forest plot of HbA1c is shown in Figure 5, 11 trails including 257 participants (cases = 129, controls = 128) examined the impact of low-ratio LA/ALA diet on the HbA1c. Our findings show no significant reduction in HbA1c levels after the low-ratio LA/ALA dietary intervention (WMD: −0.01%, 95% CI: −0.07, 0.06, *p* = 0.836). Additionally, no substantial heterogeneity among the included trials was observed (I^2^ = 0.0%, *p* = 0.465).

### 3.3. Sensitivity Analysis, Subgroup Analysis and Meta-Regression

To assess the influence of individual studies on the overall effect size, we conducted sensitivity analyses by sequentially excluding each trial from the analysis. The pooled effect size for the remaining studies, excluding the current study, is indicated by the circles in Supplementary Figures S1–S4. Sensitivity analyses revealed that the effects of the low-ratio LA/ALA diet on FBS, insulin, HOMA-IR, and HbA1c levels remained consistent regardless of the exclusion of any individual study. Upon examining Supplementary Figures S5–S8, we were visually examined to detect potential publication bias. The Begg’s test indicated no publication bias for FBS (*p* = 0.058), insulin (*p* = 0.844), HOMA-IR (*p* = 0.815), and HbA1c (*p* = 0.371). Similarly, the Egger’s test showed no publication bias for FBS (*p* = 0.149), insulin (*p* = 0.669), HOMA-IR (*p* = 0.841), and HbA1c (*p* = 0.523).

Subsequently, we conducted subgroup analyses to stratify the studies based on LA/ALA ratio (≤1, 1–5, and ≥5), region, health status, age (≤25, 25–30, and ≥30), BMI (<12 and ≥12 weeks), smoking, and duration (<12 and ≥12 weeks), as indicated in Table 2. The subgroup analyses specifically focused on the impacts of low-ratio LA/ALA diet on lowering FBS, and no significant changes were observed. These analyses showed that low-ratio LA/ALA supplementation had a significant increase on the insulin level in North America (WMD: 1.31 μIU/mL, 95% CI: 0.08, 2.54, *p* = 0.037, I^2^ = 32.0%). However, low-ratio LA/ALA supplementation had no significant effect on insulin level in Asia (WMD: 0.12 μIU/mL, 95% CI: −0.46, 0.69, *p* = 0.693, I^2^ = 0.0%) and Europe (WMD: −0.08 μIU/mL, 95% CI: −0.85, 0.69, *p* = 0.835, I^2^ = 0.0%). Studies stratified by health status showed a combined effect, showing significantly lower HbA1c level in subjects with polycystic ovary syndrome (WMD: −0.12%, 95% CI: −0.23, −0.00, *p* = 0.046, I^2^ = 0.0%). When the trials were categorized by region, the comprehensive analysis revealed a significant increase on HOMA-IR among subjects in North America (WMD: 0.47, 95% CI: 0.10, 0.84, *p* = 0.012, I^2^ = 0.0%) and an increase, but not a significant one, among subjects in Europe (WMD: 0.00, 95% CI: −0.42, 0.42, *p* = 0.260, I^2^ = 25.8%) and Asia (WMD: 0.02, 95% CI: −0.15, 0.19, *p* = 0.658, I^2^ = 0.0%).

To evaluate possible linear associations between the combined effect and continuous confounding factors (intervention duration, LA/ALA ratio, age, and BMI), we conducted meta-regressions. Our analysis revealed no significant linear relationships between FBS and intervention duration, LA/ALA ratio, age, and BMI in the included studies (intervention duration: *p* = 0.386; LA/ALA ratio: *p* = 0.781; age: *p* = 0.214; BMI: *p* = 0.732). Similar findings were observed in the meta-regressions analyzing insulin and its continuous confounders (intervention duration: *p* = 0.534; LA/ALA ratio: *p* = 0.973; age: *p* = 0.233; BMI: *p* = 0.193), as well as in the meta-regressions examining HOMA-IR and its continuous confounders (intervention duration: *p* = 0.869; LA/ALA ratio: *p* = 0.683; age: *p* = 0.713; BMI: *p* = 0.338). However, due to the limited number of included studies (less than 10), a meta-regression for HbA1c was not conducted.

### 3.4. Discussion

Despite the compelling evidence indicating the association between ALA and reduced cardiovascular disease (CVD) risk [46], as well as the lipid-lowering effects of ALA intake in hyperglycemic patients [47], the findings from meta-analyses investigating the impact of dietary ALA intake on T2DM events and glycemic control markers are inconclusive [48,49,50]. LA and ALA have the same desaturase and compete for inhibition in metabolic pathways, making the LA/ALA ratio more important than the absolute intake of both. The present systematic review and meta-analysis incorporated data from 21 RCTs encompassing 1415 participants. Our findings revealed that the administration of low-ratio LA/ALA diet did not yield any significant impact on FBS, insulin, HbA1c, and HOMA-IR. The findings were robust across the different studies included and analyzed and were not affected by sensitivity analysis.

Several studies have reported that after dietary intake of low-ratio LA/ALA, FBS decreases [32,43,51,52] or remains unchanged [30,34], and plasma insulin increases [24,37] or remains unchanged [23,29]. When conducting subgroup analysis, it was observed that individuals following a low-ratio LA/ALA diet in North America exhibited significantly higher plasma insulin and HOMA-IR levels compared to their counterparts in Asia and Europe regions. There was a trend toward lower FBS in North America and Europe, while the opposite was observed in Asia. Due to the very low quality of evidence, the meta-analysis of the trials suggests that the impact of ALA on the diagnosis of diabetes is uncertain [49]. A meta-analysis of RCTs in diabetic patients showed little effect of dietary intake of ALA on the measurement of glucose-related biomarkers [53]. A meta-analysis of low evidence suggests that ALA elevates fasting insulin levels. Higher erythrocyte ALA levels were negatively associated with T2DM risk in participants with a low genetic risk for T2DM, whereas high genetic risk eliminated the association between ALA and T2DM [54]. The variation in the response to low-ratio LA/ALA observed between Asian and Western populations could be attributed to the strong influence of genetic factors and environmental factors, including dietary habits, on the development of diabetes [55].

In the subgroup with polycystic ovary syndrome (POS), the low-ratio LA/ALA supplementation had a significant effect on the reduction of HbA1c level. Kalgaonkar’s study showed that 31 patients with POS received a diet containing 31 g of total fat per day with walnuts or almonds for 6 weeks. The walnut group reduced the low-ratio LA/ALA in the ration and plasma phospholipids, increasing insulin by 36 pmol/l and reducing HbA1c by 0.2% [22]. Another study by Vargas did not show effects on HbA1c and FBS, among others [44]. Caution should be given in interpreting these findings as the limited literature and small sample size may limit generalizability. Within this subgroup, there were only two studies, and the findings from Kalgaonkar’s study had a notable impact on the overall reliability of the pooled results.

No significant differences were observed in the outcomes across the subgroups when grouping was based on the LA/ALA ratio. In the subgroups with low-ratio LA/ALA greater than 5, results were worse for all markers of glycemic control. In the range of LA/ALA ratio less than 1, levels of FBS and Insulin were decreased but not significantly. In the subgroups with the low-ratio LA/ALA greater than 1 less than 5, levels of FBS and HbA1c were decreased but not significantly. This suggests that the high-ratio LA/ALA may have a negative impact on diabetes. These findings align with a prior systematic review, which also found that the subgroup with a n−6/n−3 PUFA ratio of less than 5 exhibited tendencies towards improvements in FBS, insulin levels, and insulin resistance [56].

When the intervention duration extended beyond 12 weeks, there was a tendency for a decrease in FBS, albeit not statistically significant. Conversely, when the intervention duration <12 weeks, the results showed the opposite trend. Despite the lack of statistical significance, it appeared that the low-ratio LA/ALA diet had a tendency to improve FBS level as compared to the high-ratio LA/ALA. This suggests that long-term treatment may have a stronger positive effect. Dietary intake of 9.5 g ALA for 6 months improved glycemic and lipid-related markers [32]. Data from 25 hypercholesterolemic menopausal women subjects suggest that a sustained long-term intake of low-ratio LA/ALA improves mild menopausal symptoms, reduces FBS and insulin levels, and facilitates changes in markers associated with cardiovascular health [52]. Dietary intake of 40 g of flaxseed for 12 weeks reduced thiobarbituric acid reactive substances (TBARS) and HOMA-IR [57]. ALA has been found to exert effects on specific diseases, which could potentially be associated with its influence on the absorption and metabolism pathways of PUFA within the body. The low-ratio LA/ALA diet initiates a cascade of beneficial effects starting with the enhancement of lipid metabolism. This improvement in lipid metabolism subsequently leads to a reduction in insulin resistance. Over a prolonged period of intervention, the low-ratio LA/ALA diet gradually contributes to the amelioration of blood glucose levels. However, given the limited amount of literature and small sample size, these findings should be interpreted with caution. Further studies in the form of large-scale, high-quality, and long-term RCTs are required to validate these results.

Most of the existing meta-analyses are mostly limited to analyzing the effect of ALA on diabetes risk and blood glucose. The current study represents a novel contribution by systematically investigating the correlation between plant-derived LA/ALA ratio and various glucose-related biomarkers. Notably, this study included high-quality summary statistics derived from a substantial sample size of 1415 subjects enrolled in 21 independent RCTs across 13 different countries. Furthermore, the robustness of the findings was supported by sensitivity analyses, and the absence of significant publication bias was demonstrated by Begg’s test and Egger’s test. However, it is important to acknowledge the limitations and shortcomings of our meta-analysis. Firstly, all the trials included in our analysis were conducted for relatively short durations, generally not exceeding 6 months. Therefore, the long-term impacts of dietary fat of the low-ratio LA/ALA on blood glucose level remain a topic that warrants further exploration in future research. Additionally, it should be noted that energy expenditure and the proportions of different types of FA were not constant across all the intervention periods in the included trials. These factors could potentially introduce variability and influence the outcomes of our analysis. It is worth noting that the FA compositions and other bioactive components can vary among different sources of LA and ALA. Thus, it remains necessary to investigate whether these factors mentioned above have an influence on the impacts of the LA/ALA ratio. Additionally, a significant proportion of the studies considered in our analysis consisted of limited sample sizes, frequently comprising less than 100 participants. Furthermore, the use of crossover (CO) designs in certain trials to bolster the effective sample size may have introduced certain complexities that could have impacted the overall outcomes. Insufficient data and imprecise categorization in certain studies might have compromised the subgroup analyses.

## 4. Conclusions

The types of FA in the diet are complex, and rarely are LA or ALA ingested singly. This systematic review and meta-analysis pooled 21 RCTs of low-ratio LA/ALA diet, encompassing 1415 subjects in 13 countries. The results found no effect of low-ratio LA/ALA diet on FBS, insulin, HOMA-IR, and HbA1c. Plasma insulin and HOMA-IR levels were significantly higher in North American patients on a low-ratio LA/ALA diet, compared to Asian and European regions. To comprehensively assess the prolonged impacts of low-ratio LA/ALA diet, it is imperative to incorporate additional RCTs encompassing diverse geographical regions and ethnicities.

## Figures and Tables

**Table 1:** Characteristics of the included studies.

Reference	Country	Participant Information	Age	BMI	Smoking	No.	M/F	Duration	Design	Low LA/ALA	High LA/ALA
Akrami 2018 [28]	Iran	Metabolic syndrome	48.6	NR	Non-smoker	52	33/19	7 W	P	0.14	19.1
Baxheinrich 2012 [29]	Netherlands	Metabolic syndrome	54.1	29.8	Mixed	163	79/84	104 W	P	4.7	29.1
Chen 2020 [30]	China	Dyslipidaemia	54.5	23.2	Mixed	243	92/151	48 W	P	7.1	30, 20
Damsgaard 2008 [31]	Denmark	Healthy	25	23.2	Mixed	33	33/0	8 W	P	4.7	7.72
Finnegan 2003 [32]	UK	Dyslipidaemia	53.7	26.1	Non-smoker	60	35/25	24 W	P	3.6	15.5
			54.5	26.2		59	35/24			1.4	15.5
Griffin 2006 [33]	UK	Healthy	59	26.3	Mixed	97	62/35	24 W	P	4.64	14
Kalgaonkar 2011 [22]	USA	Polycystic ovary syndrome	33.5	35.2	Non-smoker	31	0/31	6 W	P	4.62	22.06
Kawakami 2015 [34]	Japan	Healthy	44.5	25.1	Mixed	15	15/0	12 W	CO	1.34	9.8
Kontogianni 2013 [35]	Greece	Healthy	26	21.9	NR	37	8/29	6 W	CO	1.4	8.3
Kratz 2002 [36]	Germany	Healthy	28.9	23.8	Non-smoker	30	30/0	4 W	P	7.5	60
			22.8	22.1		25	0/25			7.5	60
			28.9	23.8		30	30/0			2.56	60
			22.8	22.1		25	0/25			2.56	60
Lee 2014 [24]	USA	Type 2 diabetes	58.6	34.5	Non-smoker	43	18/25	8 W	P	0.95	66
Ma 2010 [37]	USA	Type 2 diabetes	58.1	32.5	Non-smoker	24	10/14	8 W	P	4.48	7.75
McManus 1996 [38]	Canada	Type 2 diabetes	61.8	27.8	NR	11	8/3	12 W	CO	0.25	14.45
Minihane 2005 [39]	India	Healthy	48	26	Non-smoker	29	NR	6 W	P	9	16
Moszak 2020 [23]	Poland	Overweight or obese	48.7	39.6	Non-smoker	52	20/32	3 W	P	1.88	41.5
Nelson 2007 [40]	USA	Overweight or obese subjects	38.5	30.3	Non-smoker	57	11/46	8 W	P	1.3	10.2
Rezaei 2020 [41]	Iran	Non-alcoholic fatty liver	43.2	29.9	Mixed	68	33/35	12 W	P	0.36	228.2
Schwab 2018 [42]	Finland	Type 2 diabetes	58.9	29.2	NR	79	40/39	12 W	P	1.1	4.3
Sodergren 2001 [43]	Sweden	Dyslipidaemia	50	24.5	Mixed	19	13/6	4 W	CO	3	10
Vargas 2011 [44]	USA	Polycystic ovary syndrome	29.2	34.1	Non-smoker	34	0/34	6 W	P	1.38	9
Zhou 2019 [45]	China	Dyslipidaemia	52.7	26	Mixed	75	39/36	12 W	P	3.8, 2.05	16.04

Abbreviations: BMI, body mass index; NR, not reported; No., number of included participants; M, male; F, female; W, weeks; P, parallel; CO, crossover; LA/ALA, linoleic acid/alpha-linolenic acid.

**Table 2:** Subgroup analysis of low-ratio LA/ALA on FBS, Insulin, HbA1c, and HOMA-IR.

		FBS			Insulin			HbA1c			HOMA-IR	
Subgroup	N	WMD (95% CI)	I^2^%	N	WMD (95% CI)	I^2^%	N	WMD (95% CI)	I^2^%	N	WMD (95% CI)	I^2^%
Low-ratio LA/ALA												
≤1	3	−0.06 (−0.45, 0.33)	21.9	2	−0.17 (−0.99, 0.65)	0.0						
1–5	19	−0.00 (−0.08, 0.07)	0.0	15	0.46 (−0.27, 1.18)	13.3	7	−0.03 (−0.11, 0.05)	19.6	7	0.27 (−0.01, 0.54)	0.0
≥5	5	0.01 (−0.11, 0.13)	0.0	4	0.24 (−0.47, 0.94)	0.0	2	0.02 (−0.15, 0.19)	0.0	3	0.04 (−0.15, 0.23)	0.0
Region												
North America	6	−0.05 (−0.14, 0.02)	0.0	6	1.31 (0.08, 2.54)	32.0	5	−0.01 (−0.08, 0.07)	52.3	4	0.47 (0.10, 0.84)	0.0
Europe	13	−0.01 (−0.09, 0.07)	0.0	11	−0.08 (−0.85, 0.69)	0.0	4	−0.01 (−0.13, 0.12)	0.0	3	0.00 (−0.42, 0.42)	25.8
Asia	8	0.00 (−0.12, 0.12)	0.0	5	0.12 (−0.46, 0.69)	0.0				4	0.02 (−0.15, 0.19)	0.0
Health status												
Health	9	0.05 (−0.04, 0.15)	0.0	13	−0.35 (−1.28, 0.57)	8.6	5	−0.01 (−0.12, 0.11)	0.0	2	0.02 (−0.37, 0.41)	35.6
Dyslipidaemia	7	−0.08 (−0.18, 0.03)	7.4	17	0.35 (−0.37, 1.06)	0.0				2	0.07 (−0.12, 0.27)	0.0
Type 2 diabetes	4	−0.04 (−0.26, 0.18)	0.2	4	1.25 (−0.01, 2.60)	45.3	3	0.07 (−0.02, 0.17)	0.0	3	0.31 (−0.10, 0.71)	55.4
Overweight or obese	2	0.27 (−0.10, 0.64)	0.0	2	−0.25 (−4.36, 3.87)	0.0						
Metabolic syndrome	3	−0.18 (−0.52, 0.17)	0.0		−0.06 (−0.89, 0.76)	40.2						
Polycystic ovary syndrome	2	0.09 (−0.20, 0.38)	0.0	4	1.49 (−1.01, 3.98)	3.2	2	−0.12 (−0.23, −0.00)	0.0	2	0.42 (−0.26, 1.10)	0.0
Age												
≤45	11	0.05 (−0.04, 0.14)	0.0	10	0.01 (−0.62, 0.63)	0.0	6	−0.06 (−0.14, 0.02)	0.0	3	0.04 (−0.28, 0.36)	0.0
>45	15	−0.05 (−0.14, 0.04)	0.0	11	0.4 (−0.17, 1.05)	11.5	2	0.11 (−0.04, 0.26)	13.2	7	0.10 (−0.06, 0.26)	10.4
BMI												
≤25	9	−0.01 (−0.09, 0.07)	0.0	8	0.31 (−0.35, 0.96)	0.0	4	−0.01 (−0.13, 0.12)	0.0	2	0.07 (−0.12, 0.27)	0.0
25–30	8	0.03 (−0.12, 0.18)	2.1	4	−0.34 (−1.62, 0.95)	41.1	3	0.02 (−0.07, 0.14)	0.0	2	0.26 (−0.13, 0.65)	0.0
≥30	9	0.02 (−0.13, 0.16)	0.0	10	−0.23 (−0.41, 0.88)	2.3	3	−0.04 (−0.14, 0.06)	72.3	7	0.03 (−0.22, 0.28)	27.6
Smoking												
Non-smoker	14	0.01 (−0.10, 0.11)	0.0	13	0.19 (−0.52, 0.89)	24.3	7	−0.03 (−0.11, 0.05)	21.9	5	0.22 (−0.14, 0.59)	21.0
Mixed	9	−0.02 (−0.12, 0.08)	0.0	6	0.21 (−0.35, 0.78)	0.0				4	0.06 (−0.10, 0.22)	0.0
NR	4	0.05 (−0.11, 0.20)	0.0	3	0.16 (−2.04, 2.37)	49.9	2	0.04 (−0.08, 0.15)	0.0	2	0.07 (−0.48, 0.63)	67.1
Duration												
<12 W	15	0.01 (−0.07, 0.09)	0.0	13	0.38 (−0.37, 1.12)	32.0	7	−0.03 (−0.10, 0.05)	9.4	6	0.26 (−0.06, 0.59)	5.2
≥12 W	12	−0.02 (−0.12, 0.08)	0.0	9	0.11 (−0.42, 0.64)	0.0	2	0.04 (−0.08, 0.15)	0.0	5	0.04 (−0.12, 0.20)	0.0

Abbreviations: CI, confidential interval; N, number of included studies; LA/ALA, linoleic acid/alpha-linolenic acid; BMI, body mass index; NR, not reported; W, weeks.

## Data Availability

Data are contained within the article and Supplementary Material.

## Supplementary Materials

The supporting information can be downloaded at https://www.mdpi.com/article/10.3390/ijms241814383/s1.

## Abbreviations

LA	Linoleic acid
ALA	Alpha-linolenic acid
RCTs	Randomized controlled trials
WMD	Weighted mean difference
CI	Confidence interval
FBS	Fasting blood sugar
HOMA-IR	Homeostatic model assessment insulin resistance
HbA1c	Hemoglobin A1c
IDF	International Diabetes Federation
T2DM	Type 2 diabetes mellitus
CVD	Cardiovascular disease
POS	Polycystic ovary syndrome
EFA	Essential fatty acids
FA	Fatty acids
PUFA	Polyunsaturated fatty acid
AA	Arachidonic acid
EPA	Eicosapentaenoic acid
DHA	Docosahexaenoic acid
CLA	Conjugated linoleic acid
BMI	Body mass index
SD	Standard deviations
