# Supplementary material for: The Effect of Plant-Derived Low-Ratio Linoleic Acid/α-Linolenic Acid on Markers of Glucose Controls: A Systematic Review and Meta-Analysis
Source: Int J Mol Sci. 2023 Sep 21;24(18):14383. doi: 10.3390/ijms241814383 (PMC10532139; doi:10.3390/ijms241814383)
Supplement: Supplementary file 1 [file ijms-24-14383-s001.zip › Supplementary Figure.pdf]

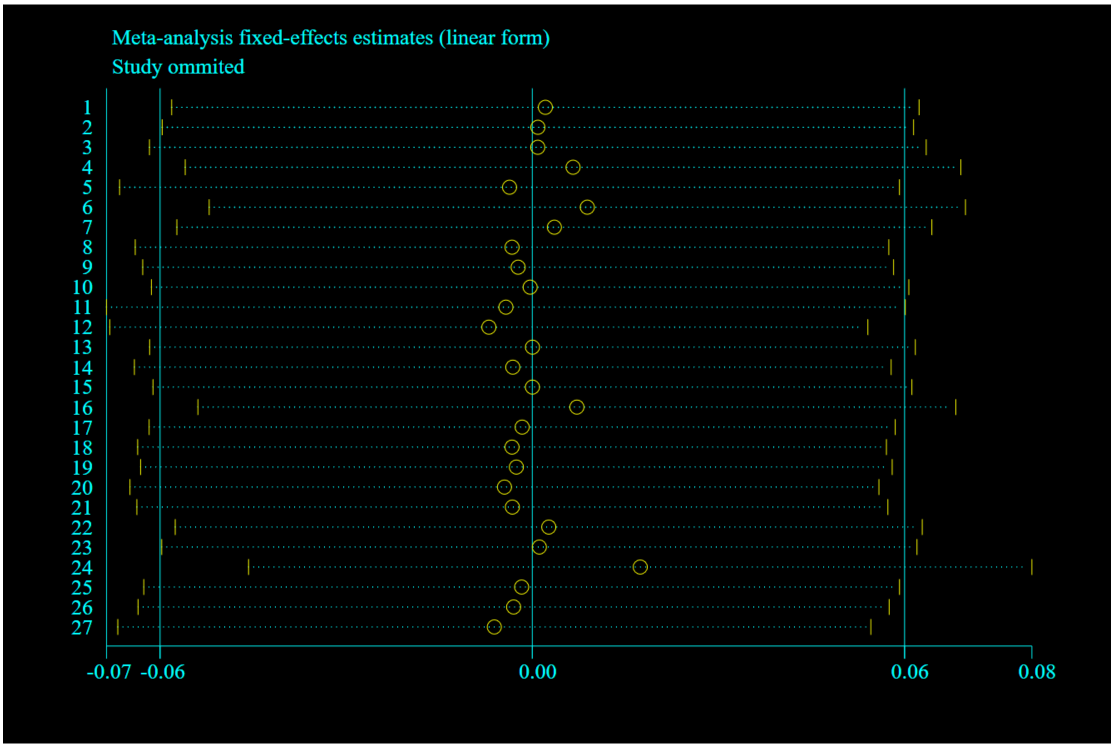

Supplementary Figure S1. Sensitivity analysis of low-ratio LA/ALA and FBS.

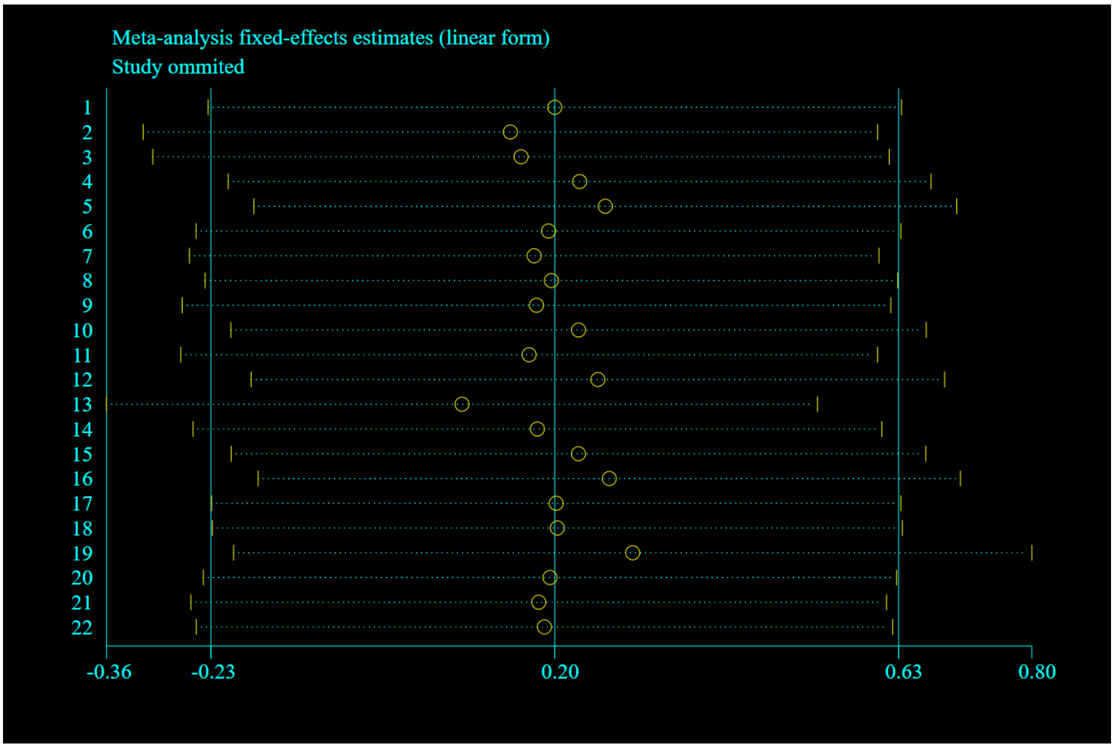

Supplementary Figure S2. Sensitivity analysis of low-ratio LA/ALA and insulin.

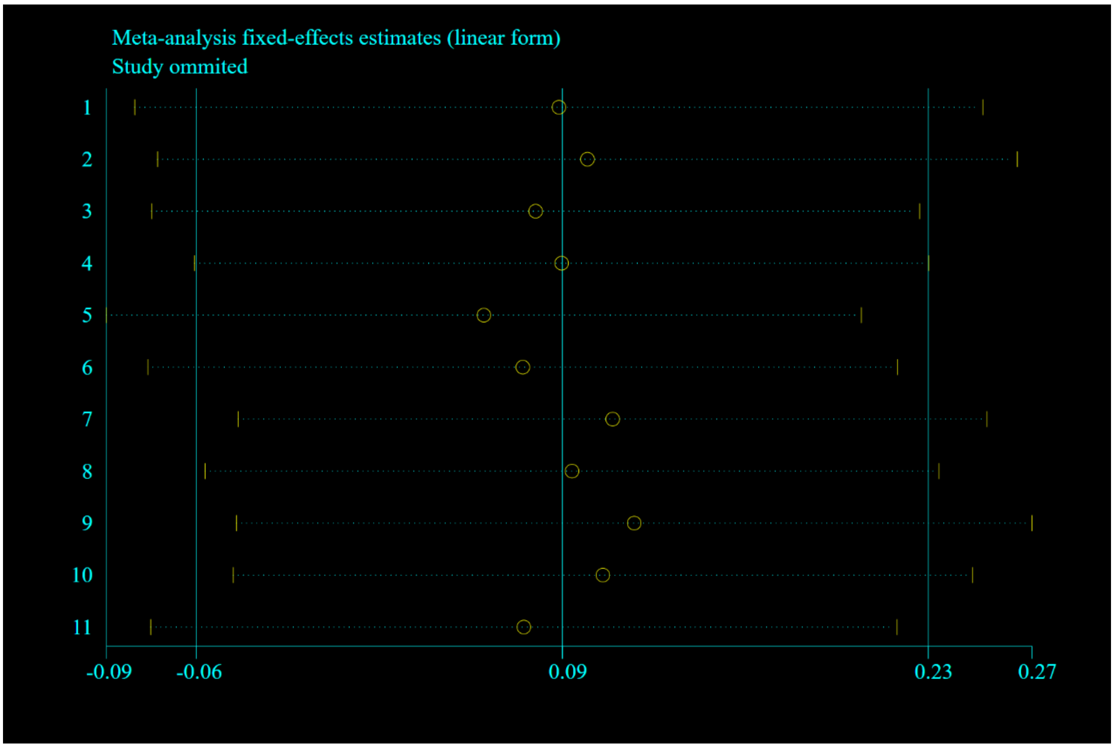

Supplementary Figure S3. Sensitivity analysis of low-ratio LA/ALA and HOMA-IR.

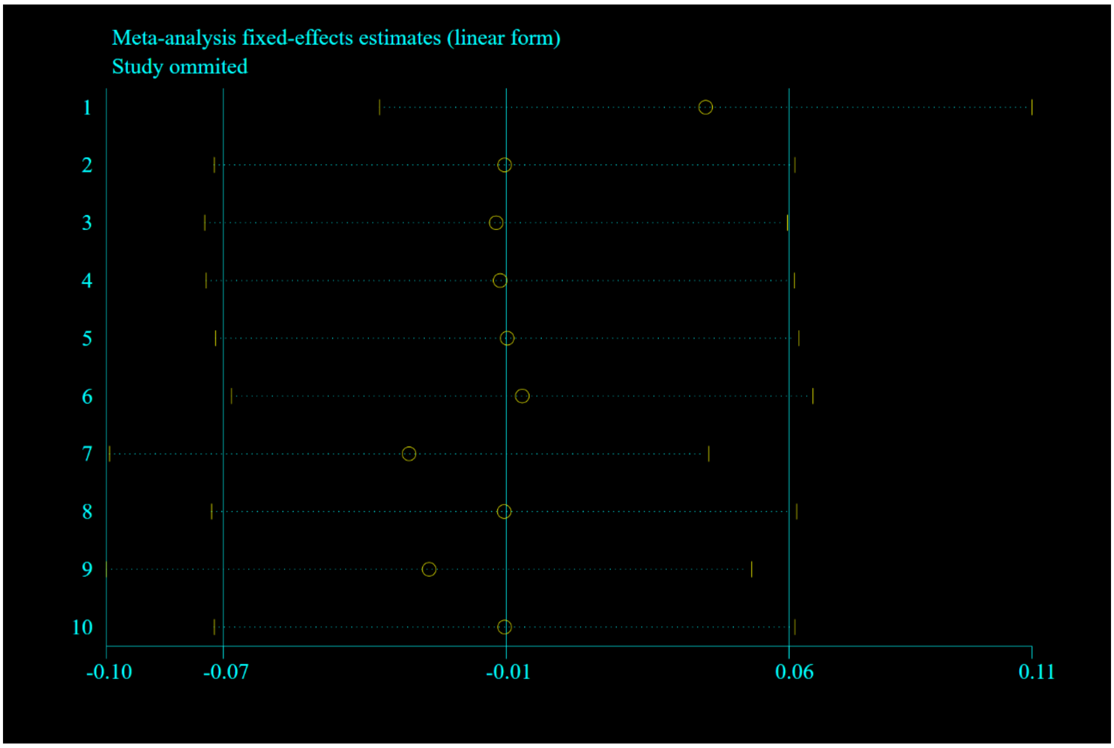

Supplementary Figure S4. Sensitivity analysis of low-ratio LA/ALA and HbA1c.

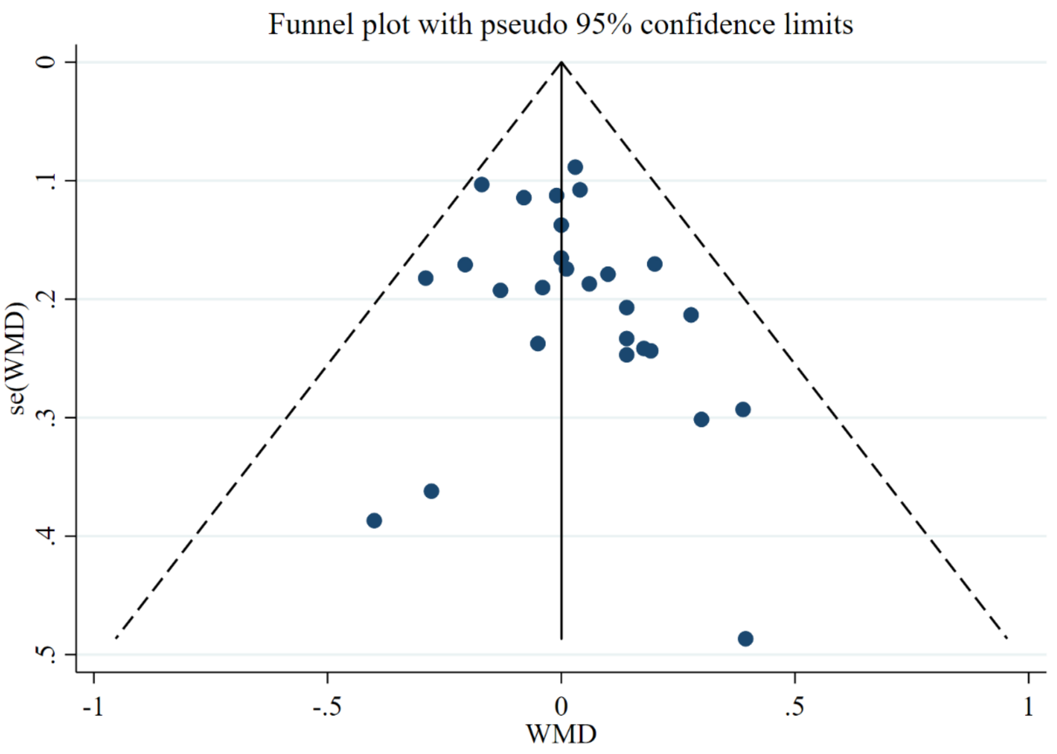

Supplementary Figure S5. Funnel plots of low-ratio LA/ALA and FBS.

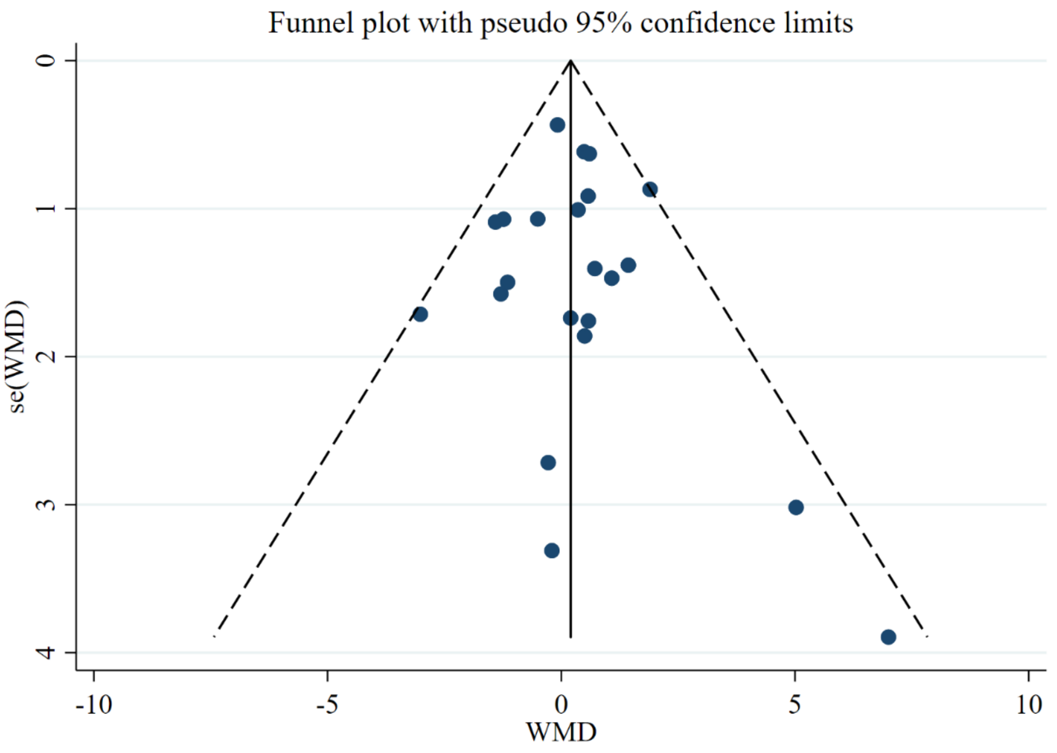

Supplementary Figure S6. Funnel plots of low-ratio LA/ALA and insulin.

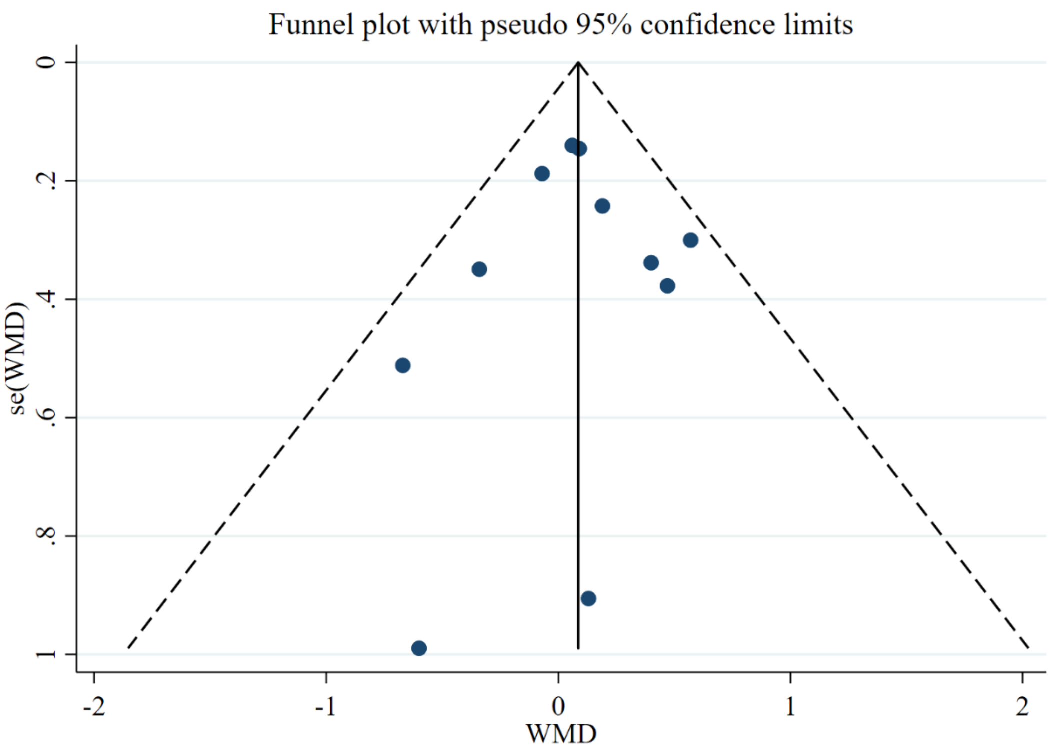

Supplementary Figure S7. Funnel plots of low-ratio LA/ALA and HOMA-IR.

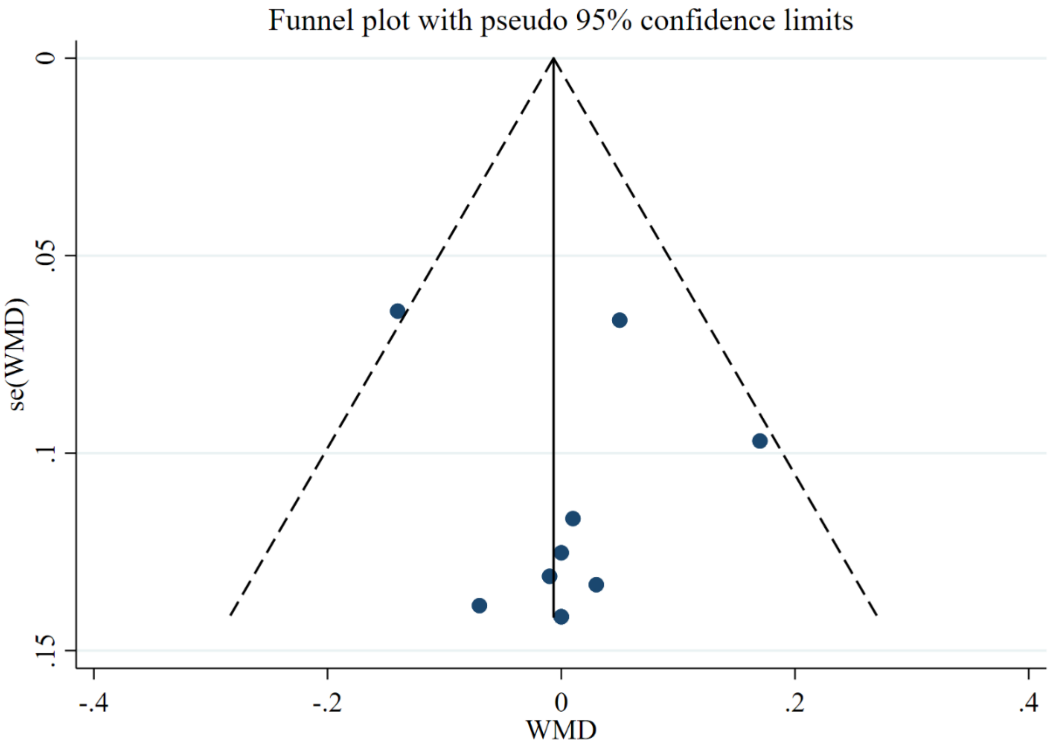

Supplementary Figure S8. Funnel plots of low-ratio LA/ALA and HbA1c.
