# Supplementary material for: The Effect of Plant-Derived Low-Ratio Linoleic Acid/α-Linolenic Acid on Markers of Glucose Controls: A Systematic Review and Meta-Analysis
Source: Int J Mol Sci. 2023 Sep 21;24(18):14383. doi: 10.3390/ijms241814383 (PMC10532139; doi:10.3390/ijms241814383)
Supplement: Supplementary file 1 [file ijms-24-14383-s001.zip › Supplementary Table.pdf]

**Supplementary Table S1.** Search strategy.

| PubMed/MEDLINE | Scopus | Web of Science | Embase |
|---|---|---|---|
| **1828** | **4726** | **3503** | **2127** |
| ("Alpha-linolenic acid"[Title/Abstract] OR "A-linolenic acid"[Title/Abstract] OR "Linoleate"[Title/Abstract] OR "Linoleic acid"[Title/Abstract] OR "Linoleate"[Title/Abstract] OR "Linoleic acid/alpha-linolenic acid"[Title/Abstract] OR "Linoleic acid/a-linolenic acid"[Title/Abstract] OR "Flaxseed"[Title/Abstract] OR "Linseed"[Title/Abstract] OR "Walnut"[Title/Abstract]) AND ("Glycosylated Hemoglobin"[Title/Abstract] OR "HbA1"[Title/Abstract] OR "HbA1c"[Title/Abstract] OR "Glycohemoglobin A"[Title/Abstract] OR "Glycated Hemoglobin"[Title/Abstract] OR "Blood Glucose"[Title/Abstract] OR "Blood Sugar"[Title/Abstract] OR Hyperglycemia[Title/Abstract] OR Glycemia[Title/Abstract] OR HOMA*[Title/Abstract] OR "homeostatic model for insulin resistance"[Title/Abstract] OR "fasting blood insulin"[Title/Abstract] OR FBI[Title/Abstract] OR FBG [Title/Abstract] OR | ( TITLE-ABS-KEY ( "Glycated Hemoglobin A" ) OR TITLE-ABS-KEY ( "Blood Glucose" ) OR TITLE-ABS-KEY ( "Hyperglycemia" ) OR TITLE-ABS-KEY ( "Insulin Resistance" ) OR TITLE-ABS-KEY ( "Hyperinsulinism" ) OR TITLE-ABS-KEY ( "Diabetes Mellitus" ) OR TITLE-ABS-KEY ( "Glycosylated Hemoglobin" ) OR TITLE-ABS-KEY ( "HbA1" ) OR TITLE-ABS-KEY ( "Hb A1c" ) OR TITLE-ABS-KEY ( "Glycohemoglobin A" ) OR TITLE-ABS-KEY ( "Glycated Hemoglobin" ) OR TITLE-ABS-KEY ( "Blood Sugar" ) OR TITLE-ABS-KEY ( glycemia ) OR TITLE-ABS-KEY ( "HOMA" ) OR TITLE-ABS-KEY ( "homeostatic model for insulin resistance" ) OR TITLE-ABS-KEY ( "fasting blood insulin" ) OR TITLE-ABS-KEY ( "FBI" ) OR TITLE-ABS-KEY ( "FBG" ) OR TITLE-ABS-KEY ( "FBS" ) OR TITLE-ABS-KEY ( "Insulin Sensitivity" ) OR TITLE-ABS-KEY ( insulin ) OR TITLE-ABS-KEY ( hyperinsulin ) OR TITLE-ABS-KEY ( "Oral Glucose Tolerance Test" ) OR TITLE-ABS-KEY ( "OGTT" ) AND TITLE-ABS-KEY ( "alpha-linolenic acid" ) OR TITLE-ABS- | TS=("Alpha-linolenic acid" OR "A-linolenic acid" OR "Linoleate" OR "Linoleic acid" OR "Linoleate" OR "Linoleic acid/alpha-linolenic acid" OR "Linoleic acid/a-linolenic acid" OR "Flaxseed" OR "Linseed" OR "Walnut") AND TS=( "Glycated Hemoglobin A" OR "Blood Glucose" OR "Hyperglycemia" OR "Insulin Resistance" OR "Hyperinsulinism" OR "Diabetes Mellitus" OR "Glycosylated Hemoglobin" OR "HbA1" OR "Hb A1c" OR "Glycohemoglobin A" OR "Glycated Hemoglobin" OR "Blood Sugar" OR glycemia OR "HOMA" OR "homeostatic model for insulin resistance" OR "fasting blood insulin" OR "FBI" OR "FBG" OR "FBS" OR "Insulin Sensitivity" OR insulin OR "insulin*" OR | ('alpha-linolenic acid':ab,ti OR 'a-linolenic acid':ab,ti OR 'linoleic acid':ab,ti OR 'linoleate':ab,ti OR 'linoleic acid/alpha-linolenic acid':ab,ti OR 'linoleic acid/a-linolenic acid':ab,ti OR 'flaxseed':ab,ti OR 'linseed':ab,ti OR 'walnut':ab,ti) AND ('glycated hemoglobin a':ab,ti OR 'blood glucose':ab,ti OR 'hyperglycemia':ab,ti OR 'insulin resistance':ab,ti OR 'hyperinsulinism':ab,ti OR 'diabetes mellitus':ab,ti OR 'glycosylated hemoglobin':ab,ti OR 'hba1':ab,ti OR 'hb a1c':ab,ti OR 'glycohemoglobin a':ab,ti OR 'glycated hemoglobin':ab,ti OR 'blood sugar':ab,ti OR glycemia:ab,ti OR 'homa':ab,ti OR 'homeostatic model for insulin resistance':ab,ti OR 'fasting blood insulin':ab,ti OR 'fbi':ab,ti OR |

| | | | |
|---|---|---|---|
| FBS[Title/Abstract] OR "Insulin Resistance"[Title/Abstract] OR "Insulin Sensitivity"[Title/Abstract] OR "Hyperinsulinemia"[Title/Abstract] OR Hyperinsulinism[Title/Abstract] OR Insulin [Title/Abstract] OR Hyperinsulin [Title/Abstract] OR "Oral Glucose Tolerance Test"[Title/Abstract] OR OGTT[Title/Abstract] OR "Diabetes Mellitus"[Title/Abstract]) | KEY ( "A-linolenic acid" ) OR TITLE-ABS-KEY ( "Linoleate" ) OR TITLE-ABS-KEY ( "Linoleic acid" ) OR TITLE-ABS-KEY ( "Linoleic acid/alpha-linolenic acid" ) OR TITLE-ABS-KEY ( "Linoleic acid/a-linolenic acid" ) OR TITLE-ABS-KEY ( "Flaxseed" ) OR TITLE-ABS-KEY ( "Linseed" ) OR TITLE-ABS-KEY ( "Walnut" ) ) | "hyperinsulin*" OR "Oral Glucose Tolerance Test" OR "OGTT") | 'fbg':ab,ti OR 'fbs':ab,ti OR 'insulin sensitivity':ab,ti OR insulin:ab,ti OR 'insulin*':ab,ti OR 'hyperinsulin*':ab,ti OR 'oral glucose tolerance test':ab,ti OR 'ogtt':ab,ti) |

**Supplementary Table S2.** Dietary intakes of nutrient of the participants at intervention period.

| Reference | Group | Energy (kcal/d) | Protein | Carbohydrate | Fat | SFA | MUFA | PUFA | Total LA | Total ALA | LA and ALA Source |
|---|---|---|---|---|---|---|---|---|---|---|---|
| | | | (% of total energy intake/d) | | | | | | | | |
| Akrami2018 | Low LA/ALA | 2481 | 15.1 | 56.3 | 28.4 | 7.5 | 3.6 | 17.3[b] | 2.1[a] | 15.2[b] | 25 ml flaxseed oil |
| | High LA/ALA | 2490 | 14.8 | 56.2 | 28.8 | 7.6 | 4.7 | 16.5[a] | 15.68[b] | 0.82[a] | 25 ml sunflower oil |
| Bemelmans2002 | Low LA/ALA | 2796.3 | | | 37.7 | 13.5 | 12.1 | 12.1 | 9.5 | 2.3[b] | ALA-rich margarine |
| | High LA/ALA | 2796.3 | | | 37.7 | 13.5 | 12.1 | 12.1 | 10.6 | 0.4[a] | LA-rich margarine |
| Chen2020 | Low LA/ALA | 2248.2 | 16.3 | 52.0 | 31.7 | 7.2 | 11.9 | 7.5 | 7.1 | 1.0[b] | 27 g blend oil |
| | High LA/ALA | 2161.4 | 16.1 | 53.4 | 30.5 | 7.4 | 11.3 | 6.8 | 6.3 | 0.21[a] | 27 g peanut oil |
| | High LA/ALA | 2072.3 | 15.5 | 54.6 | 29.8 | 7.2 | 9.4 | 8.4 | 7.8 | 0.39[a] | 27 g corn oil |
| Damsgaard2008 | Low LA/ALA | 2722 | 15 | 48 | 36 | 12.2 | 13.2 | 5[a] | 4.07[a] | 0.86 | 10.3 g (0.0-52.5g/d) rapeseed oil |
| | High LA/ALA | 2654.6 | 14 | 51 | 35 | 10.5 | 11.5 | 7.5[b] | 6.54[b] | 0.85 | 10.3 g (0.0-52.5g/d) sunflower oil |
| Finnegan2003 | Low LA/ALA | 2342.2[a] | 15.5 | 45.3 | 36 | 11.5 | 10.1 | 8.4 | 6.22[a] | 1.73[b] | 25 g ALA margarine (rapeseed and linseed oils) |
| | Low LA/ALA | 2485.6[ab] | 15.4 | 43.7 | 37.8 | 12.3 | 10.9 | 8.8 | 4.74[a] | 3.44[c] | 25 g ALA margarine (rapeseed and linseed oils) |
| | High LA/ALA | 2891.9[b] | 16.1 | 43.3 | 36.2 | 11.9 | 10.5 | 8.2 | 7.13[b] | 0.47[a] | 25 g LA margarine (sunflower and safflower oils) |
| Griffin2006 | Low LA/ALA | 2093.6 | 16.4 | 42.3 | 36.1 | 11.5 | 14.2 | 6.8[a] | 5.1[a] | 1.1[b] | 32 g rapeseed oil |
| | High LA/ALA | 2148.6 | 16.4 | 41.0 | 37.4 | 12.2 | 14.4 | 8.2[b] | 7[b] | 0.5[a] | 32 g high-oleic sunflower oil |
| Kalgaonkar2011 | Low LA/ALA | 1541.1[a] | 17 | 45 | 40 | 18.69[b] | 11.10[a] | 10.66[b] | 8.76[b] | 1.90[b] | 31 g walnut-oil |
| | High LA/ALA | 1718.2[b] | 19 | 44 | 39 | 15.19[a] | 18.33[b] | 4.11[a] | 3.93[a] | 0.18[a] | 31 g almond-oil |
| Kawakami2015 | Low LA/ALA | 2261.4 | 13.02 | 55.75 | 25.51 | | | | 8.11[a] | 6.03[b] | 10 g flaxseed oil |
| | High LA/ALA | 2289.8 | 13.33 | 53.51 | 28.42 | | | | 15.68[b] | 1.6[a] | 10 g corn oil |
| Kontogianni2013 | Low LA/ALA | 1929.2 | 15.9 | 44.2 | 37.0 | 12.3 | 14.5[a] | 8.4[b] | | 4.06[b] | 15 ml flaxseed oil |

| | | | | | | | | | | | |
|---|---|---|---|---|---|---|---|---|---|---|---|
| | High LA/ALA | 1806.1 | 18.4 | 47.6 | 39.0 | 12.0 | 17.6[b] | 6.0[a] | | 0.40[a] | 15 ml olive oil |
| Kratz2002 | Low LA/ALA | 2318.3 | 14.4 | 46.9 | 38.8 | 10.7 | 23.3[b] | 3.4[a] | 3[a] | 0.4[a] | Olive oil diet |
| | Low LA/ALA | 2574.0 | 14.3 | 47.3 | 38.4 | 9.1 | 19[b] | 8.9[b] | 6.4b | 2.5[b] | Rapeseed oil diet |
| | High LA/ALA | 2538.2 | 14.2 | 47.6 | 38.2 | 10 | 8.7[a] | 18.4[c] | 18.1[c] | 0.3[a] | Sunflower oil diet |
| Lee2014 | Low LA/ALA | | | | | | | | 1.8[a] | 1.9[b] | 7 echium oil capsules and 3 borage oil capsules |
| | High LA/ALA | | | | | | | | 3.96[b] | 0.06[a] | 9 corn oil capsules |
| Ma2010 | Low LA/ALA | 1765 | 17 | 39 | 45[b] | 10.88 | 9.18 | 16.83[b] | 13.26[b] | 2.96[b] | 56 g of shelled, unroasted English walnuts |
| | High LA/ALA | 1685 | 19 | 43 | 38[a] | 12.52 | 9.61 | 4.91[a] | 3.31[a] | 0.43[a] | Ad libitum diet without walnuts |
| McManus1996 | Low LA/ALA | | | | | | | | 14.2% of total fatty caids | 57.5% of total fatty caids | 35 mg/kg body weight flaxseed oil |
| | High LA/ALA | | | | | | | | 13% of total fatty caids | 0.9% of total fatty caids | 35 mg/kg body weight olive oil |
| Minihane2005 | Low LA/ALA | | | | 39 | 9 | 15[b] | 5.7[a] | 5[a] | 0.7 | olive oil |
| | High LA/ALA | | | | 37 | 10 | 10[a] | 10.7[b] | 10[b] | 0.7 | coin oil |
| Moszak2020 | Low LA/ALA | | 20% | 50-55% | 25-30% | | | | 4.2[a] | 2.24[b] | 20 ml rapeseed oil |
| | High LA/ALA | | 20% | 50-55% | 25-30% | | | | 9.98[b] | 0.24[a] | 20 ml amaranth seed oil |
| Nelson2007 | Low LA/ALA | 1924.3 | | | 38.6 | 10.6 | 13.1 | 12.1 | 6.8 | 5.3[b] | Subjects increased ALA intake to 5% of total energy via flaxseed oil capsule. Subjects were advised to decrease their intake of high-fat foods, whole and 2% milk products, and added fats. Subjects |
| | High LA/ALA | 2062.9 | | | 35.8 | 12.2 | 13.8 | 7.1 | 6.12 | 0.6[a] | |

| | | | | | | | | | | | |
|---|---|---|---|---|---|---|---|---|---|---|---|
| | | | | | | | | | | | were also provided lists of lower-fat alternatives to these foods. |
| Rezaei2020 | Low LA/ALA | 1807 | 66.30 | 13.17 | 20.77 | 6.18 | 5.58 | 8.82 | $3.62^{a}$ | $9.96^{b}$ | 20 g flaxseed oil |
| | High LA/ALA | 1781 | 63.83 | 13.32 | 22.99 | 6.52 | 6.77 | 9.75 | $12.68^{b}$ | $0.044^{a}$ | 20 g sunflower oil |
| Schwab2018 | Low LA/ALA | 2215.5 | $15.6^{a}$ | $38.2^{a}$ | $42.5^{b}$ | 12.1 | 15.0 | $11.6^{b}$ | $5.48^{b}$ | $5.04^{b}$ | 27 g camelina sativa oil and canola oil |
| | High LA/ALA | 1938.5 | $17.7^{b}$ | $42.5^{b}$ | $34.0^{a}$ | 11.3 | 13.1 | $5.6^{a}$ | $4.18^{a}$ | $0.97^{a}$ | 27 g canola oil and olive oil |
| Sodergren2001 | Low LA/ALA | 2127.1 | 14.8 | 47.3 | 35.9 | $10.3^{a}$ | $15.7^{b}$ | $7.5^{b}$ | $5.4^{b}$ | $1.8^{b}$ | Rapeseed oil provide 27% of total energy |
| | High LA/ALA | 2318.3 | 14.4 | 47.5 | 36.2 | $17.6^{b}$ | $10.9^{a}$ | $4.6^{a}$ | $4.0^{a}$ | $0.4^{a}$ | Butter and olive oil provide 27% of total energy |
| Vargas2011 | Low LA/ALA | 1735 | 16.9 | 48.2 | 34.8 | | | 4.6 | $2.66^{a}$ | $1.93^{b}$ | 6 capsules flaxseed oil |
| | High LA/ALA | 1735 | 16.9 | 48.2 | 34.8 | | | 3.91 | $3.51^{b}$ | $0.39^{a}$ | 6 capsules soybean oil |
| Zhou2019 | Low LA/ALA | 1927 | | | 33.72 | 7.89 | 10.13 | 11.44 | 7.85 | $3.82^{c}$ | 25 ml flaxseed oil and corn oil |
| | Low LA/ALA | 1917 | | | 33.57 | 8.12 | 10.42 | 11.60 | 8.92 | $2.35^{b}$ | 25 ml flaxseed oil and corn oil |
| | High LA/ALA | 1960 | | | 34.35 | 8.17 | 11.53 | 10.7 | 9.87 | $0.62^{a}$ | 25 ml corn oil |

SFA, saturated fatty acids; MUFA, monounsaturated fatty acids; PUFA, polyunsaturated fatty acids; LA, linoleic acid; ALA, $\alpha$-linolenic acid; Different letters indicate significant differences ($p < 0.05$) between each group, and the same letters indicate that there is no significant difference ($p > 0.05$) between each group.

**Supplementary Table S3.** Quality assessment of the included studies.

| Study | Random Sequence Generation | Allocation Concealment | Blinding of Participants and Personnel | Blinding of Outcome Assessments | Incomplete Outcome Data | Selective Outcome Reporting | Other Bias |
|---|---|---|---|---|---|---|---|
| Akrami2018 | L | U | U | U | L | L | L |
| Bemelmans2002 | L | L | L | U | L | U | L |
| Chen2020 | L | L | L | U | L | U | L |
| Damsgaard 2008 | L | L | L | L | L | L | L |
| Finnegan2003 | L | U | L | U | L | U | L |
| Griffin2006 | U | U | U | U | U | U | L |
| Kalgaonkar2011 | L | L | L | L | L | L | L |
| Kawakami 2015 | U | U | L | U | L | U | L |
| Kontogianni 2013 | L | L | H | U | L | U | L |
| Kratz2002 | U | U | U | U | U | U | U |
| Lee 2014 | U | U | H | U | L | U | L |
| Ma 2010 | U | U | L | L | L | L | L |
| McManus1996 | L | U | L | L | L | U | L |
| Minihane2005 | U | U | L | U | L | U | L |
| Moszak2020 | U | U | L | L | L | U | L |
| Nelson2007 | U | U | U | L | L | L | L |
| Rezaei2020 | L | L | L | U | L | L | L |
| Schwab2018 | L | U | U | L | L | U | L |
| Sodergren2001 | U | U | U | U | U | U | L |
| Vargas2011 | U | U | L | U | U | U | U |
| Zhou2019 | U | U | L | U | L | L | L |

H: high risk of bias; U: unclear risk of bias; L: low risk of bias.
